# Early parental death and being not in education, employment, or training (NEET-status) in Norway: a population-wide study on the moderating role of parental education

**DOI:** 10.1093/eurpub/ckaf081

**Published:** 2025-07-08

**Authors:** Lamija Delalic, Jonathan Wörn, Bjørn-Atle Reme

**Affiliations:** Knowledge Department, Norwegian Labour and Welfare Administration, Oslo, Norway; Centre for Fertility and Health, Norwegian Institute of Public Health, Oslo, Norway; Centre for Fertility and Health, Norwegian Institute of Public Health, Oslo, Norway; Department of Health Management and Health Economics, University of Oslo, Oslo, Norway

## Abstract

Childhood parental death has been linked to adverse young adult outcomes, potentially influenced by family background. This study quantifies the association between parental death during childhood and NEET-status (not in education, employment, or training) in young adulthood, focusing on the moderating role of parental education. Causes of death were leveraged to explore the extent of confounding in the relationship between parental death and NEET-status. The study utilized Norwegian registry data from birth cohorts 1977–87 (574 229 individuals). We identified individuals with and without the experience of parental death between ages 0–17 and tracked their NEET-status between ages 22–29. Poisson regression models estimated incidence risk ratios for NEET years based on parental death, parental education, their interaction, and control variables. To address confounding, causes of death were categorized as more exogenous (i.e. neoplasms) or more endogenous (e.g. suicide or drug-related deaths). Early parental death and lower parental education were both linked to more years in NEET status. Incidence risk ratios varied by cause of death, ranging from 1.19 for neoplasms [95% confidence interval (CI): 1.13–1.25] to 2.36 for drug-related causes (95% CI: 2.17–2.56). Lower parental education amplified the association between NEET-status and parental death from most causes, but to the smallest extent for neoplasms. The association between parental death and NEET status was stronger among individuals with parents with lower parental education. When the cause of death was unrelated to parental education, the modifying effect of parental education was smaller, suggesting that stronger associations in low-education families may largely reflect confounding factors.

## Introduction

Adverse childhood experiences are associated with adverse outcomes in adolescence and early adulthood, including mental health issues, lower educational attainment, and reduced labour market participation [1–8]. Early parental death is an example of such adversity, affecting children both directly, through emotional distress and grief, and indirectly, through its effect on other family members who hold an important supporting role [8]. Moreover, there are often negative impacts on household income and the remaining parent’s ability and capacity to parent effectively. These combined effects can have long-term impacts on a child’s health and life trajectory.

The transition into early adulthood, marked by educational pursuits and entry into the labour market, is important for a range of long-term outcomes, such as health, financial stability, social integration, and family formation [9–11]. Notably, NEET-status (**N**ot in **E**ducation, **E**mployment, or **T**raining) has emerged as an indicator of societal disengagement among young adults [12]. Hence, understanding the factors contributing to NEET-status is critical to develop policies that can limit the risk of young adults entering a trajectory that leads to economic, health, and social marginalization later in life.

Families differ along dimensions that affect both the risk of experiencing parental death and its impact on the remaining family members. For example, children of highly educated or high-income parents face a lower risk of losing a parent during childhood [13]. In addition, families with high parental education or income may have access to resources that soften the impact of a traumatic experience [14]. The cause of parental death may also affect children’s outcomes, and these causes are not evenly distributed across socioeconomic groups. For example, deaths due to drug abuse or suicide—often preceded by a history of mental illness—are more prevalent in disadvantaged families, while early deaths from illnesses such as cancer are more evenly distributed [13, 15, 16].

Several studies have documented the association between parental death during childhood and outcomes as a young adult, including differences by socioeconomic status [14, 17, 18]. However, much of the evidence leaves questions about mechanisms unanswered. In this study, we examine how the likelihood of different causes of parental death varies by parental education. We then examine the association between parental death and NEET-status, stratified both by parental education and cause of parental death. Since the degree of confounding varies by cause, this approach helps assess the role of confounding in the association between parental death and NEET-status, as well as the potential buffering role of parental education.

## Methods

### Study population

This population-based cohort study is based on all individuals born between 1977 and 1987 and registered in the Norwegian Population Register. The register contains information about gender, year of birth, year of death, and identity of family members. Using pseudonymized identification numbers, other population-wide administrative registers were linked, including The National Education Database, the National Registry for Personal Taxpayers, and the Cause of Death Registry. The study was approved, and participant consent was waived by the Regional Committee for Medical and Health Research Ethics South-East Norway (REK, approval 2018/434).

### Early parental death

Early parental death was the exposure and was retrieved from the Population Register. Causes of death were retrieved from the Norwegian Cause of Death Register, originally coded using the International Classification of Diseases. However, the available data were classified according to the European Shortlist for Causes of Death, Version 1998. See Supplementary Table S1 for the full list and details of the categorization applied in this study. The age at which the parents had died (if applicable) was calculated as the difference between parental year of death and offspring birth year. Individuals were considered as exposed if they had experienced parental death between age 0–17, and as the control group otherwise. Provided the aim of the study was to examine the association between losing a parent during childhood and early adult NEET status, we excluded individuals who lost a parent before birth.

### Not in education, employment, or training status

The outcome was NEET-status: neither employed, engaged in education, nor participating in vocational training. NEET-status was operationalized as follows: a binary variable coded as 1 if (i) income was below the National Insurance scheme basic amount (1G), corresponding to approximately USD 10 000 per year, and (ii) the individual was not registered in education or vocational training, according to the national education database. There was one outcome registration per individual per year during ages 22–29. Years prior to age 22 were excluded because common non-NEET activities in these age groups are not captured by our measure of NEET-status. This refers specifically to compulsory military training of all boys [19], legally required to enlist for military service, typically beginning at age 19 or 20 and lasting 12 months, unless unfit for service due to health problems, and the high share of Norwegians taking a gap year immediately upon completing upper secondary school [20].

### Parental education level

Parental education level was retrieved from the National Education Database, measured at child’s age of five. Parental education level was dichotomized based on whether at least one parent had completed a bachelor’s degree or higher, corresponding to levels 6 or higher on the International Standard Classification of Education.

### Statistical analysis

We present the number of predicted years in NEET-status for combinations of the experience of early parental death and different causes of parental death, respectively, with parental education level. These were predicted using Poisson regression models, with an eight-year follow-up period, estimating the number of predicted years in NEET-status between ages 22 and 29. We further present incidence rate ratios [IRRs with 95% confidence intervals (CIs)] for number of years in NEET-status. Poisson models of NEET-years included parental education level, the occurrence or cause of parental death, and their interaction. All models adjust for migration background (six categories indicating own and parental migration history, see Table 1), and potential time trends or birth cohort effects by including a dummy for each birth year. Robust standard errors were used in all regression models. The analyses were performed using Stata 16.1. The study adheres to the main principles of the STROBE guidelines for cohort studies.

**Table 1.** Descriptive statistics^a^

		Highest parental education	
	Total	No university	Any university
*N*	574 229	389 415	184 814
Years observed, mean (SD)	7.9 (0.8)	7.9 (0.7)	7.8 (0.9)
Early parental death (age 17 or before)	18 339 (3.2%)	13 862 (3.6%)	4477 (2.4%)
Cause of parental death			
No early parental death	555 890 (96.8%)	375 553 (96.4%)	180 337 (97.6%)
Drug-related	1001 (0.2%)	886 (0.2%)	115 (0.1%)
Suicide or self-harm	2052 (0.4%)	1586 (0.4%)	466 (0.3%)
External causes of injury	2606 (0.5%)	2120 (0.5%)	486 (0.3%)
Other diseases	5654 (1.0%)	4491 (1.2%)	1,163 (0.6%)
Neoplasms	6393 (1.1%)	4275 (1.1%)	2,118 (1.1%)
Unknown cause	633 (0.1%)	504 (0.1%)	129 (0.1%)
Deceased parent is father	13 161 (71.8%)	10 244 (73.9%)	2917 (65.2%)
Child sex is male	294 408 (51.3%)	199 681 (51.3%)	94 727 (51.3%)
Child age at parental death, mean (SD)	10.8 (4.8)	10.7 (4.8)	11.1 (4.7)
Child year of birth, mean (SD)	1982.0 (3.2)	1982.0 (3.2)	1982.2 (3.2)
Child migration background			
No immigrant background	506 468 (88.2%)	348 926 (89.6%)	157 542 (85.2%)
First-generation immigrant	19 509 (3.4%)	14 128 (3.6%)	5381 (2.9%)
Second-generation immigrant	9037 (1.6%)	6580 (1.7%)	2457 (1.3%)
Foreign-born with one Norwegian parent	3778 (0.7%)	1807 (0.5%)	1971 (1.1%)
Norwegian-born with one foreign-born parent	28 465 (5.0%)	14 839 (3.8%)	13 626 (7.4%)
Born abroad to Norwegian parents	6972 (1.2%)	3135 (0.8%)	3837 (2.1%)

a Rounding error hides educational differences in the share of unknown causes of death. The shares with unknown cause of parental death are 0.13% for children with parents that do not have university education and 0.07% for children with parents that do have university education.

## Results

Our analytical sample consisted of *N* = 574 229 individuals, who contributed with an average of 7.9 [standard deviation (SD) = 0.8] years to the analysis (4 507 749 person-years). Of this, 51.3% were male, and 32.2% had at least one parent with university education (Table 1). Further, 18 339 (3.2%) lost one of their parents at age 17 or before, with a higher share observed among those without a parent with university education (3.6% vs. 2.4%, *P* < .001). Neoplasms were the most common cause of early parental death, and the only cause that was equally common among individuals from parents with and without university education (*P* = .10; all other causes: *P* < .001). From an initial sample of 575 389, we excluded those who experienced the death of both parents before age 18 (*n* = 282), because living without any parent at young ages is arguably a markedly different experience than having a surviving parent, and those whose parent died in the calendar year before the individual’s birth (*n* = 60). A total of *n* = 818 individuals were excluded because they were outside the country or not alive in all years. See Table 1 for further details.

### Incidence of NEET status by early parental death and parental level of education

**Figure 1.**
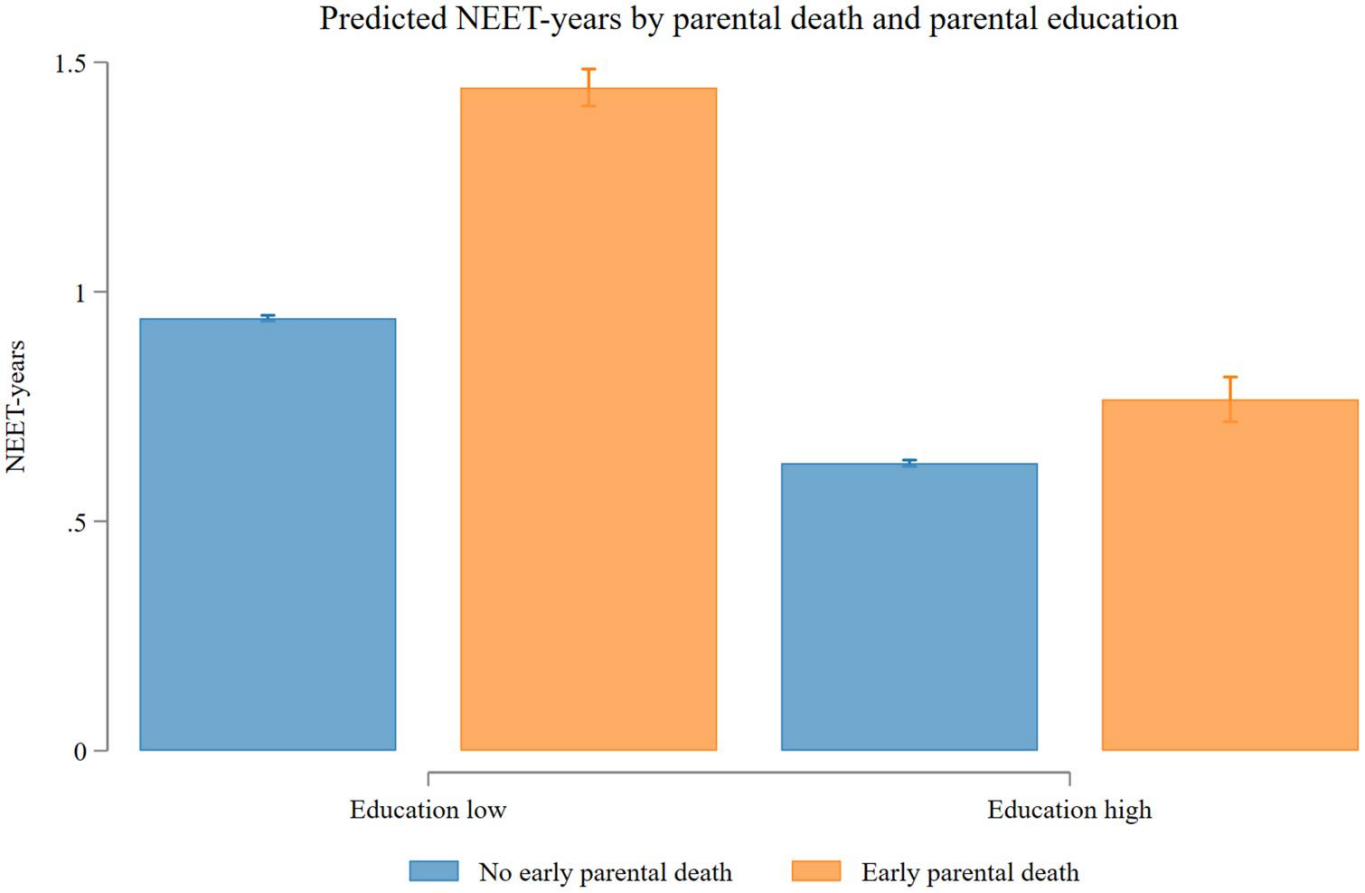
Estimated number of years in NEET-status by early parental death and parental education, age 22–29. The number of NEET-years between ages 22 and 29 was estimated using Poisson regression. NEET-years were regressed on the interaction between a binary indicator of early parental death and parental education level. Robust standard errors were used. The model was then used to predict the number of NEET-years, keeping migration background and birth year fixed at observed values. See Model 2 in Supplementary Table S2.

NEET-status was associated with parental education level and parental death (Fig. 1). At both education levels, the predicted number of NEET-years was higher among individuals who experienced early parental death (no university: 1.44 years; 95% CI: 1.40–1.49; university: 0.77 years; 95% CI: 0.72–0.81) compared to those who did not (no university: 0.94 years; 95% CI: 0.94–0.95; university: 0.63 years; 95% CI: 0.62–0.63). Figure 1 further illustrates that the number of NEET-years varied with educational level, both in the group with and the group without the experience of early parental death. The number of NEET-years among individuals from families ‘without’ university education and ‘without’ experiencing parental death was comparable to individuals from families ‘with’ university education and ‘with’ the experience of early parental death.

### Incidence of NEET status by education level, parental loss, and cause of death

**Figure 2.**
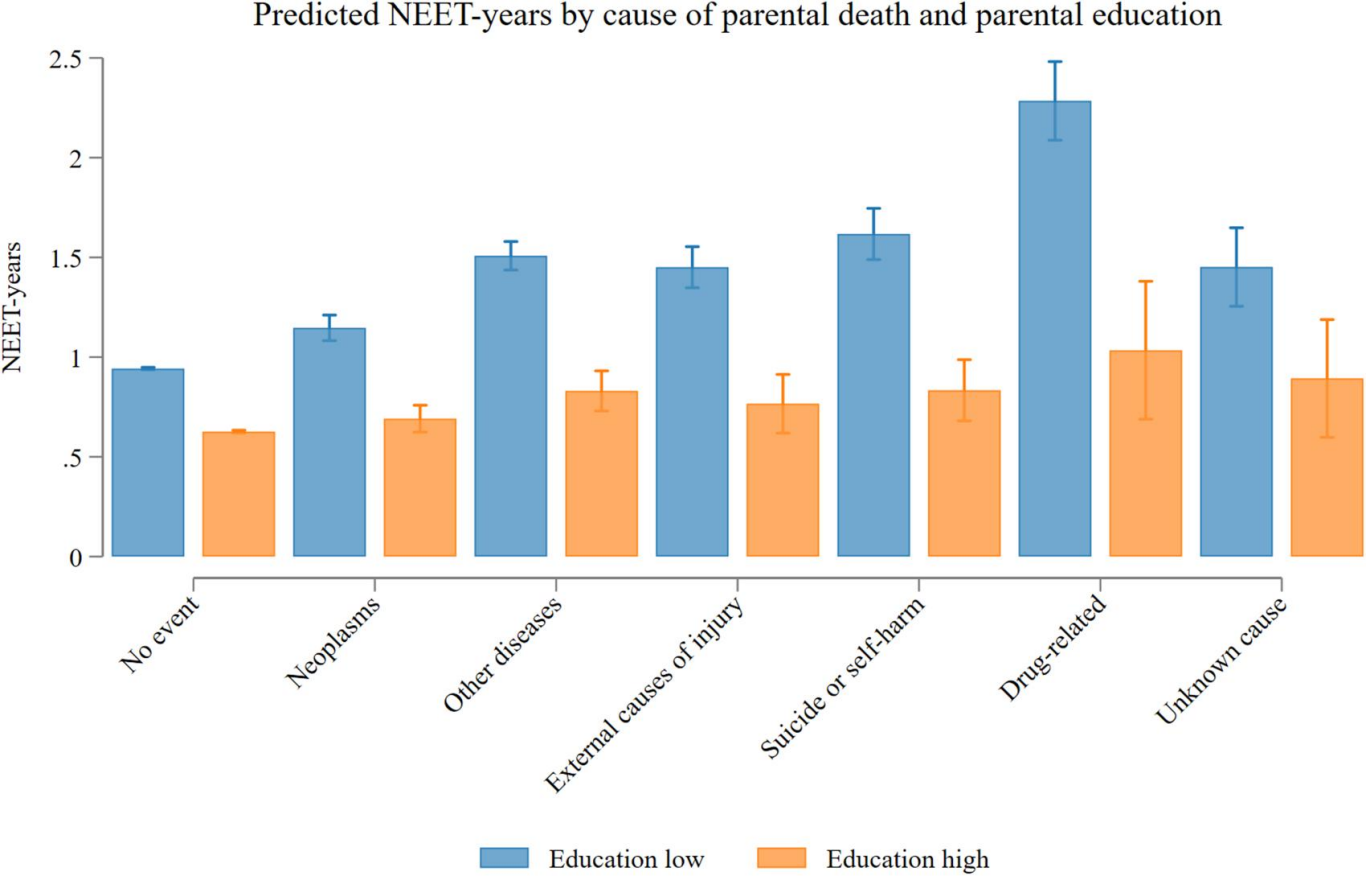
Estimated number of years in NEET-status across cause of parental death and parental education, age 22–29. The number of NEET-years between ages 22 and 29 was estimated using Poisson regression. NEET-years were regressed on the interaction between binary indicators of cause of early parental death and parental education level. Robust standard errors were used. The model was then used to predict the number of NEET-years, keeping migration background and birth year fixed at observed values. See Model 2 in Table 2.

There were significant differences in the number of NEET-years across causes of death and parental education level (Fig. 2). For individuals with parents ‘without’ university education, the number of predicted NEET-years ranged from 1.15 (95% CI: 1.08–1.21) for those who experienced early parental death from neoplasms to 2.28 (95% CI: 2.09–2.48) for those who experienced early parental death due to drug-related causes. For individuals with parents ‘with’ university education, the corresponding numbers ranged from 0.69 (95% CI: 0.62–0.76) for those who experienced early parental death to neoplasms and 1.03 (95% CI: 0.69–1.38) for those who experienced early parental death due to drug-related causes.

Complementing the descriptive analyses, we report IRRs to compare the relative difference in incidence of NEET-years across causes of parental death, and by levels of parental education. The models account for year of birth and migration background. Model 1 (Table 2) shows for the total sample that neoplasms are associated with an IRR of 1.19 (95% CI: 1.13–1.25). A higher incidence rate was observed for external causes of injury (IRR = 1.50; 95% CI: 1.40–1.60), other diseases (IRR = 1.56; 95% CI: 1.49–1.63), and suicide or self-harm (IRR = 1.65; 95% CI: 1.53–1.78). The highest incidence rate was observed for parental death due to drug-related causes (IRR = 2.36; 95% CI: 2.17–2.56). Models 2 and 3 include an interaction term for cause of parental death with lower and higher parental education, respectively. These models show that the incidence rate for NEET-years in the case of parental death due to neoplasms does not differ (CI overlapping 1) for individuals from parents with and without university education. The models predict a higher incidence rate among individuals with lower parental education for all other known causes of parental death. The most important finding of this analysis is that the association between neoplasms—the only cause of early parental death equally common across educational groups—with NEET-status does not differ by parental education, while the association of all other causes—which are more common in individuals from families without university education—with NEET-status are stronger in those with lower education backgrounds.

**Table 2.** IRR from Poisson regression for NEET-years between ages 22 and 29 on parental cause of death, parental education, and potential confounding variables^a^

	M1	M2 interaction	M3 interaction
		Reference: Any university	Reference: No university
No university (M2)	1.51	1.50	Reference
	[1.49–1.53]	[1.49–1.52]	
Any university (M3)	Reference	Reference	0.66
			[0.66–0.67]
Cause of death			
No early parental death	Reference	Reference	Reference
Drug-related	2.36	1.65	2.42
	[2.17–2.56]	[1.18–2.31]	[2.22–2.64]
Suicide or self-harm	1.65	1.33	1.72
	[1.53–1.78]	[1.11–1.60]	[1.58–1.86]
External causes of injury	1.50	1.22	1.54
	[1.40–1.60]	[1.01–1.48]	[1.43–1.65]
Other diseases	1.56	1.33	1.60
	[1.49–1.63]	[1.17–1.50]	[1.53–1.68]
Neoplasms	1.19	1.10	1.22
	[1.13–1.25]	[1.00–1.22]	[1.15–1.29]
Unknown cause	1.52	1.43	1.54
	[1.34–1.73]	[1.02–1.98]	[1.34–1.76]
Cause of death # education (M2/M3)			
Drug-related #		1.47	0.68
No/any university		[1.04–2.07]	[0.48–0.96]
Suicide or self-harm #		1.29	0.78
No/any university		[1.05–1.58]	[0.63–0.95]
External causes of injury #		1.26	0.79
No/any university		[1.03–1.55]	[0.65–0.97]
Other diseases #		1.21	0.83
No/any university		[1.06–1.38]	[0.73–0.94]
Neoplasms #		1.10	0.91
No/any university		[0.98–1.23]	[0.81–1.02]
Unknown cause #		1.08	0.93
No/any university		[0.76–1.54]	[0.65–1.32]
Constant	0.08	0.08	0.11
	[0.07–0.08]	[0.07–0.08]	[0.11–0.12]
Individuals	574 229	574 229	574 229

a All models account for year of birth and migration background.

Additional analyses show that results are practically identical when using Negative Binomial regression instead of Poisson regression (Supplementary Table S3). Using the education of the surviving mother (father) in the case of paternal (maternal) death confirms the strongest association with NEET-status for drug-related causes and the weakest association for neoplasms irrespective of parental sex (referring to known causes of death; Supplementary Table S4). For both sexes, the largest (smallest) educational differences are found for drug-related causes (neoplasms). Results are largely comparable for girls and boys, but wide CIs limit the opportunities to detect potential gender differences (Supplementary Table S5).

## Discussion

In this observational study of 574 229 individuals born between 1977 and 1987, we found that the death of a parent during childhood was associated with an increased incidence of NEET-status during young adulthood (ages 22–29). This association varied significantly across causes of parental death and parental education levels.

Most causes of parental death were more frequent among individuals from families without university education than among their counterparts from families with university education. The exception were deaths due to neoplasms, one of the most common causes of early parental death, where the likelihood of the event was equal (1.1%) and the strength of the association with later NEET-status was very similar across parental education levels. The strongest associations between parental death and NEET-status—and the most pronounced social gradients in these associations—were observed for causes of death more common in lower-socioeconomic status (SES) families. These causes, among others drug abuse or external causes, are more likely to reflect pre-existing disadvantages that influence both the likelihood of parental death and later NEET-status. Such differences may include differences in parental mental health, parental health behaviours, health literacy, and other lifestyle or environmental differences [21]. To summarize, the differences in the association between early parental death and later NEET status across parental education levels are driven by causes of death where confounding likely is more pronounced.

### Comparison to previous studies

Earlier studies on the impact of parental death during childhood have found that both psychological, educational, and labour market impacts were stronger for external causes of death (accidents, violent deaths, suicide, or drug-related) than natural causes (disease-related) [5, 22–25]. This study extends the existing literature along several dimensions. First, while most prior studies have focused on short-term impacts, in the years following the parental death, this study examines long term impacts by following the cohorts from the early event, between age 0–17, with outcome measured in young adulthood (ages 22–29). Second, although previous studies have adjusted for socioeconomic position, we are unaware of population-wide studies that have investigated how potential impacts differ across a broad range of causes of death.

Our findings align with previous studies that do not find a moderating effect of socioeconomic status [14, 26, 27]. It also complements these studies by showing that while associations are stronger for some causes of death, such as drug-related, the SES differences in the overall association likely stems from a combination of confounding and a higher prevalence of more traumatic causes of death in lower SES families. Our study, to some extent, contrasts recent evidence based on Finnish register data, finding that a highly educated surviving parent buffered effects of parental loss in the case of natural causes of death (as opposed to external causes) [25]. Our study also complements these findings by providing a larger sample, longer time period, and more fine-grained set of causes of death. The more detailed data on causes of death allows for separating more clearly based on whether confounding is to be expected, compared to a relatively crude division into natural or external causes.

To summarize, provided the substantial attenuation of associations for neoplasms, our study suggests that previously reported associations including all types of causes of parental deaths cannot be interpreted causally due to confounding. For example, poor parental mental health may both contribute to parental suicide and that the children have mental health issues, and struggle in school and working life.

### Interpretation and relevance

In terms of implications, our study suggests that children from families with less resources are more at risk of longer-term negative impacts following early parental death. The analysis of different causes of parental death raises the question to which extent confounding drives this pattern, because NEET-risk does not differ by parental education for causes of death that are equally distributed by educational family background.

While our study was conducted in Norway, a country with a wide-reaching welfare state that mitigates social inequality, it highlights critical links between childhood trauma and later life outcomes that are likely to exist in various contexts. Thus, our results may inform policy initiatives beyond Norway. At the same time, differences across countries matter. For example, the significant disparities observed across familial socioeconomic backgrounds suggest that social gradients could be even more pronounced in countries with weaker social support systems, suggesting an even stronger need for policies that assist children from low SES backgrounds in navigating and overcoming adversity.

### Strengths and weaknesses

The main strength of the study is that it covers 11 full birth cohorts during a 30-year period, with complete registrations of educational activity, labour market attachment, family relations, and causes of death. Hence, the risk of sample selection or recall bias is minor.

The study has several limitations. Firstly, while we account for parental education, migration background, and birth cohort, we do not explicitly account for parental income, household structure, or other factors that could influence both the likelihood of early parental death and the risk of becoming NEET, potentially introducing bias. Moreover, the study uses a dichotomization of parental education, which could oversimplify the role of education as a moderating factor. Another limitation is that, while parental death generally is a traumatic experience, the degree of trauma varies, which our study cannot account for. More specifically, external causes (suicide or violent deaths) may be less exogenous, but they could also be a more traumatic experience, either due to their sudden nature (e.g. traffic accidents) or long period of neglect prior to death (e.g. drug-related deaths). Such heterogeneity in trauma is also true for cancers, as there are considerable differences in the length from diagnosis to the eventual death. To summarize, although comparing impacts across causes of death is interesting as there is more or less confounding (or randomness), the differences in impacts across causes of death may to some extent be attributed to differences in trauma related to their difference in nature. An additional source of variation stems from differences in support provided to next of kin of diseased family members. In Norway, the support for family members of individuals with cancer is considered broad and encompassing, which might contribute to small educational differences in NEET-status following parental death from neoplasms. At the same time, it should be noted that good follow-up cannot completely shield children from the psychological impact of losing a parent [28].

## Conclusion

Our results confirm that there are substantial differences in the incidence of NEET-status as young adult across parental background, both among children that experience parental death and those that do not. However, the differential impact across parental education level might be largely due to confounding, indicating that while children from families with low levels of parental education are more likely to experience parental death, the causal impact may not differ substantially.

## Supplementary Material

ckaf081_Supplementary_Data

## Data Availability

The data used in this study contains individual-level information on causes of death, family ties, education level, and labour market attachment for entire cohorts of the Norwegian population. These data were made available on loan for research purposes. Other researchers may apply for access to the same sources—see helsedata.no/en and https://ssb.no/en/data-til-forskning-utlan-av-data-tilforskere. Key points Early parental death and lower parental education are linked to more years in NEET-status in Norway. Incidence risk ratios varied by cause of death, ranging from 1.19 for neoplasms to 2.36 for drug-related causes. Lower parental education amplified the association between NEET-status and parental death, but to a little degree for neoplasms. Stronger associations in low-education families may reflect confounding factors. Children from families with less resources are more at risk of longer-term negative impacts following early parental death.
