# Supplementary material for: Early parental death and being not in education, employment, or training (NEET-status) in Norway: a population-wide study on the moderating role of parental education
Source: Eur J Public Health. 2025 Jul 8;35(5):889–95. doi: 10.1093/eurpub/ckaf081 (PMC12529293; doi:10.1093/eurpub/ckaf081)
Supplement: ckaf081_Supplementary_Data [file ckaf081_supplementary_data.docx]

**Supplementary Materials**

Supplementary Table 1. EU Shortlist Causes of Death - V.1998

| Level | Code | Description | ICD-10 code | Recoded |
| --- | --- | --- | --- | --- |
| 1 | 1 | Infectious and parasitic diseases | A00-B99 | Other diseases |
| 2 | 2 | Tuberculosis | A15-A19, B90 | Other diseases |
| 2 | 3 | Meningococcal infection | A39 | Other diseases |
| 2 | 4 | AIDS (HIV-disease) | B20-B24 | Other diseases |
| 2 | 5 | Viral hepatitis | B15-B19 | Other diseases |
| 1 | 6 | Neoplasms | C00-D48 | Neoplasms |
| 2 | 7 | Malignant neoplasms | C00-C97 | Neoplasms |
| 3 | 8 | of which Malignant neoplasm of lip, oral cavity, pharynx | C00-C14 | Neoplasms |
| 3 | 9 | of which Malignant neoplasm of oesophagus | C15 | Neoplasms |
| 3 | 10 | of which Malignant neoplasm of stomach | C16 | Neoplasms |
| 3 | 11 | of which Malignant neoplasm of colon | C18 | Neoplasms |
| 3 | 12 | of which Malignant neoplasm of rectum and anus | C19-C20-C21 | Neoplasms |
| 3 | 13 | of which Malignant neoplasm liver and the intrahepaticbile ducts | C22 | Neoplasms |
| 3 | 14 | of which Malignant neoplasm of pancreas | C25 | Neoplasms |
| 3 | 15 | of which Malignant neoplasm of larynx and trachea/bronchus/lung | C32-C34 | Neoplasms |
| 3 | 16 | of which Malignant melanoma of skin | C43 | Neoplasms |
| 3 | 17 | of which Malignant neoplasm of breast | C50 | Neoplasms |
| 3 | 18 | of which Malignant neoplasm of cervix uteri | C53 | Neoplasms |
| 3 | 19 | of which Malignant neoplasm of other parts of uterus | C54-55 | Neoplasms |
| 3 | 20 | of which Malignant neoplasm of ovary | C56 | Neoplasms |
| 3 | 21 | of which Malignant neoplasm of prostate | C61 | Neoplasms |
| 3 | 22 | of which Malignant neoplasm of kidney | C64 | Neoplasms |
| 3 | 23 | of which Malignant neoplasm of bladder | C67 | Neoplasms |
| 3 | 24 | of which Malignant neoplasm of lymph./haematopoietic tissue | C81-C96 | Neoplasms |
| 1 | 25 | Diseases of the blood(-forming organs), immunol. disorders | D50-D89 | Other diseases |
| 1 | 26 | Endocrine, nutritional and metabolic diseases | E00-E90 | Other diseases |
| 2 | 27 | Diabetes mellitus | E10-E14 | Other diseases |
| 1 | 28 | Mental and behavioural disorders | F00-F99 | Other diseases |
| 2 | 29 | Alcohol abuse (including alcoholic psychosis) | F10 | Drug-related |
| 2 | 30 | Drug dependence, toxicomania | F11-F16, F18-F19 | Drug-related |
| 1 | 31 | Diseases of the nervous system and the sense organs | G00-H95 | Other diseases |
| 2 | 32 | Meningitis (other than 03) | G00-G03 | Other diseases |
| 1 | 33 | Diseases of the circulatory system | I00-I99 | Other diseases |
| 2 | 34 | Ischaemic heart diseases | I20-I25 | Other diseases |
| 2 | 35 | Other heart diseases | I30-I33, I39-I52 | Other diseases |
| 2 | 36 | Cerebrovascular diseases | I60-I69 | Other diseases |
| 1 | 37 | Diseases of the respiratory system | J00-J99 | Other diseases |
| 2 | 38 | Influenza | J10-J11 | Other diseases |
| 2 | 39 | Pneumonia | J12-J18 | Other diseases |
| 2 | 40 | Chronic lower respiratory diseases | J40-J47 | Other diseases |
| 3 | 41 | of which asthma | J45-J46 | Other diseases |
| 1 | 42 | Diseases of the digestive system | K00-K93 | Other diseases |
| 2 | 43 | Ulcer of stomach, duodenum and jejunum | K25-K28 | Other diseases |
| 2 | 44 | Chronic liver disease | K70, K73-K74 | Other diseases |
| 1 | 45 | Diseases of the skin and subcutaneous tissue | L00-L99 | Other diseases |
| 1 | 46 | Diseases of the musculoskeletal system/connective tissue | M00-M99 | Other diseases |
| 2 | 47 | Rheumatoid arthritis and osteoarthrosis | M05-M06, M15-M19 | Other diseases |
| 1 | 48 | Diseases of the genitourinary system | N00-N99 | Other diseases |
| 2 | 49 | Diseases of kidney and ureter | N00-N29 | Other diseases |
| 1 | 50 | Complications of pregnancy, childbirth and puerperium | O00-O99 | Other diseases |
| 1 | 51 | Certain conditions originating in the perinatal period | P00-P96 | Other diseases |
| 1 | 52 | Congenital malformations and chromosomal abnormalities | Q00-Q99 | Other diseases |
| 2 | 53 | Congenital malformations of the nervous system | Q00-Q07 | Other diseases |
| 2 | 54 | Congenital malformations of the circulatory system | Q20-Q28 | Other diseases |
| 1 | 55 | Symptoms, signs, abnormal findings, ill-defined causes | R00-R99 | Other diseases |
| 2 | 56 | Sudden infant death syndrome | R95 | Other diseases |
| 2 | 57 | Unknown and unspecified causes | R96-R99 | Other diseases |
| 1 | 58 | External causes of injury and poisoning | V01-Y89 | External causes |
| 2 | 59 | Accidents | V01-X59 | External causes |
| 3 | 60 | of which Transport accidents | V01-V99 | External causes |
| 3 | 61 | of which Accidental falls | W00-W19 | External causes |
| 3 | 62 | Accidental poisoning | X40-X49 | Drug-related |
| 2 | 63 | Suicide and intentional self-harm | X60-X84 | Suicide or self-harm |
| 2 | 64 | Homicide, assault | X85-Y09 | External causes |
| 2 | 65 | Events of undetermined intent | Y10-Y34 | External causes |

Supplementary Table 2. IRR from Poisson-regression for NEET-years between ages 22 and 29 on early parental death, parental education, and potential confounding variables.

|  | **Model 1** | **Model 2** |
| --- | --- | --- |
| No university (ref. any) | 1.52 | 1.50 |
|  | [1.50,1.54] | [1.49,1.52] |
|  |  |  |
| Early parental death (ref. no) | 1.48 | 1.22 |
|  | [1.44,1.52] | [1.15,1.30] |
|  |  |  |
| No university # Early parental death |  | 1.25 |
|  |  | [1.17,1.35] |
|  |  |  |
| Constant | 0.07 | 0.08 |
|  | [0.07,0.08] | [0.07,0.08] |
| Observations | 574,229 | 574,229 |

Note: All models account for year of birth and migration background.

Supplementary Table 3.1. IRR from Negative Binomial-regression for NEET-years between ages 22 and 29 on parental death/cause of death, parental education, and potential confounding variables. Compare to Model 1 and Model 2 in Supplementary Table 2.

|  | **Model 1** | **Model 2** |
| --- | --- | --- |
| No university (ref. any) | 1.52 | 1.50 |
|  | [1.50,1.54] | [1.49,1.52] |
|  |  |  |
| Early parental death (ref. no) | 1.48 | 1.22 |
|  | [1.44,1.52] | [1.15,1.30] |
|  |  |  |
| No university # Early parental death |  | 1.25 |
|  |  | [1.17,1.35] |
|  |  |  |
| Constant | 0.07 | 0.08 |
|  | [0.07,0.08] | [0.07,0.08] |
| Observations | 574,229 | 574,229 |

Note: All models account for year of birth and migration background.

Supplementary Table 3.2. IRR from Negative Binomial-regression for NEET-years between ages 22 and 29 on cause of death, parental education, and potential confounding variables. Compare to Model 1 and Model 2 in Table 2.

|  | **M1** | **M2 Interaction**: |
| --- | --- | --- |
|  |  | Reference:  any university |
| No university (ref. any) | 1.53 | 1.52 |
|  | [1.51,1.55] | [1.50,1.54] |
|  |  |  |
| *Cause of death* |  |  |
| No early parental death | reference | reference |
|  |  |  |
| Drug-related | 2.36 | 1.64 |
|  | [2.17,2.57] | [1.16,2.31] |
|  |  |  |
| Suicide or self-harm | 1.67 | 1.38 |
|  | [1.55,1.80] | [1.15,1.67] |
|  |  |  |
| External causes of injury | 1.50 | 1.26 |
|  | [1.41,1.61] | [1.04,1.53] |
|  |  |  |
| Other diseases | 1.57 | 1.34 |
|  | [1.50,1.64] | [1.18,1.51] |
|  |  |  |
| Neoplasms | 1.19 | 1.11 |
|  | [1.13,1.25] | [1.00,1.22] |
|  |  |  |
| Unkown cause | 1.55 | 1.42 |
|  | [1.36,1.77] | [1.03,1.97] |
|  |  |  |
| ***Cause of death # education (M2)*** |  |  |
| Drug-related # |  | 1.50 |
| No university |  | [1.05,2.14] |
|  |  |  |
| Suicide or self-harm # |  | 1.27 |
| No university |  | [1.04,1.56] |
|  |  |  |
| External causes of injury # |  | 1.24 |
| No university |  | [1.01,1.52] |
|  |  |  |
| Other diseases # |  | 1.22 |
| No university |  | [1.07,1.39] |
|  |  |  |
| Neoplasms # |  | 1.10 |
| No university |  | [0.98,1.24] |
|  |  |  |
| Unkown cause # |  | 1.12 |
| No university |  | [0.78,1.59] |
|  |  |  |
| Constant | 0.08 | 0.08 |
|  | [0.07,0.08] | [0.07,0.08] |
| alpha | 4.28 | 4.28 |
|  | [4.25,4.31] | [4.25,4.31] |
| Individuals | 574,229 | 574,229 |

Note: Models account for year of birth and migration background.

Supplementary Table 4. IRR from Poisson-regression for NEET-years between ages 22 and 29 on parental cause of death, parental education, and potential confounding variables. The model for paternal education includes children without experience of parental death and with experience of maternal death. The model for maternal education includes children without experience of parental death and with experience of paternal death. Compare to Model 2 in Table 2.

|  | Model 2 | Model 2 |
| --- | --- | --- |
|  | (Father’s education) | (Mother’s education) |
| No university (ref. any) | 1.45 | 1.51 |
|  | [1.43,1.47] | [1.49,1.54] |
|  |  |  |
| *Cause of death* |  |  |
| No early parental death | reference | reference |
|  |  |  |
| Drug-related | 1.60 | 1.72 |
|  | [0.66,3.87] | [1.12,2.66] |
|  |  |  |
| Suicide or self-harm | 1.11 | 1.51 |
|  | [0.73,1.67] | [1.17,1.96] |
|  |  |  |
| External causes of injury | 1.12 | 1.30 |
|  | [0.67,1.86] | [1.01,1.66] |
|  |  |  |
| Other diseases | 1.19 | 1.31 |
|  | [0.85,1.68] | [1.10,1.56] |
|  |  |  |
| Neoplasms | 1.03 | 1.22 |
|  | [0.87,1.22] | [1.03,1.43] |
|  |  |  |
| Unkown cause | 0.55 | 1.43 |
|  | [0.19,1.57] | [0.91,2.25] |
|  |  |  |
| ***Cause of death # education*** |  |  |
| Drug-related # | 1.68 | 1.41 |
| No university | [0.68,4.16] | [0.91,2.20] |
|  |  |  |
| Suicide or self-harm # | 1.39 | 1.16 |
| No university | [0.88,2.18] | [0.88,1.52] |
|  |  |  |
| External causes of injury # | 1.50 | 1.18 |
| No university | [0.88,2.56] | [0.91,1.54] |
|  |  |  |
| Other diseases # | 1.42 | 1.22 |
| No university | [0.99,2.03] | [1.02,1.46] |
|  |  |  |
| Neoplasms # | 1.12 | 1.02 |
| No university | [0.93,1.35] | [0.86,1.22] |
|  |  |  |
| Unkown cause # | 2.67 | 1.11 |
| No university | [0.86,8.25] | [0.69,1.78] |
|  |  |  |
| Constant | 0.08 | 0.07 |
|  | [0.07,0.08] | [0.07,0.07] |
| Individuals | 561,068 | 569,051 |

Note: Models account for year of birth and migration background.

Supplementary Table 5. IRR from Poisson-regression (separately for boys and girls) for NEET-years between ages 22 and 29 on parental cause of death, parental education, and potential confounding variables. Compare to Model 2 in Table 2.

|  | Model 2 | Model 2 |
| --- | --- | --- |
|  | (Girls) | (Boys) |
| No university (ref. any) | 1.67 | 1.36 |
|  | [1.64,1.70] | [1.33,1.38] |
|  |  |  |
| *Cause of death* |  |  |
| No early parental death | 1.56 | 1.68 |
|  | [0.90,2.70] | [1.10,2.56] |
| Drug-related |  |  |
|  | 1.40 | 1.27 |
|  | [1.07,1.82] | [0.98,1.65] |
| Suicide or self-harm |  |  |
|  | 1.21 | 1.24 |
|  | [0.92,1.59] | [0.94,1.62] |
| External causes of injury |  |  |
|  | 1.32 | 1.33 |
|  | [1.11,1.56] | [1.12,1.58] |
| Other diseases |  |  |
|  | 1.22 | 1.01 |
|  | [1.06,1.40] | [0.88,1.15] |
| Neoplasms |  |  |
|  | 1.56 | 1.31 |
|  | [1.01,2.43] | [0.80,2.16] |
| Unkown cause | 1.56 | 1.68 |
|  | [0.90,2.70] | [1.10,2.56] |
|  |  |  |
| ***Cause of death # education*** |  |  |
| Drug-related # | 1.41 | 1.60 |
| No university | [0.80,2.47] | [1.03,2.48] |
|  |  |  |
| Suicide or self-harm # | 1.15 | 1.44 |
| No university | [0.86,1.53] | [1.09,1.91] |
|  |  |  |
| External causes of injury # | 1.20 | 1.32 |
| No university | [0.89,1.60] | [0.99,1.77] |
|  |  |  |
| Other diseases # | 1.18 | 1.24 |
| No university | [0.99,1.42] | [1.03,1.49] |
|  |  |  |
| Neoplasms # | 1.00 | 1.21 |
| No university | [0.85,1.17] | [1.04,1.42] |
|  |  |  |
| Unkown cause # | 0.94 | 1.23 |
| No university | [0.58,1.52] | [0.72,2.09] |
|  |  |  |
| Constant | 0.08 | 0.07 |
|  | [0.07,0.08] | [0.07,0.08] |
| Individuals | 279,821 | 294,408 |

Note: Models account for year of birth and migration background.
